# Machine-learning to characterise neonatal functional connectivity in the preterm brain

**DOI:** 10.1016/j.neuroimage.2015.08.055

**Published:** 2016-01-01

**Authors:** G. Ball, P. Aljabar, T. Arichi, N. Tusor, D. Cox, N. Merchant, P. Nongena, J.V. Hajnal, A.D. Edwards, S.J. Counsell

**Affiliations:** Centre for the Developing Brain, Division of Imaging Sciences & Biomedical Engineering, King's College London, London, United Kingdom

**Keywords:** Brain development, fMRI, Prematurity, Support vector machines

## Abstract

Brain development is adversely affected by preterm birth. Magnetic resonance image analysis has revealed a complex fusion of structural alterations across all tissue compartments that are apparent by term-equivalent age, persistent into adolescence and adulthood, and associated with wide-ranging neurodevelopment disorders. Although functional MRI has revealed the relatively advanced organisational state of the neonatal brain, the full extent and nature of functional disruptions following preterm birth remain unclear.

In this study, we apply machine-learning methods to compare whole-brain functional connectivity in preterm infants at term-equivalent age and healthy term-born neonates in order to test the hypothesis that preterm birth results in specific alterations to functional connectivity by term-equivalent age.

Functional connectivity networks were estimated in 105 preterm infants and 26 term controls using group-independent component analysis and a graphical lasso model. A random forest–based feature selection method was used to identify discriminative edges within each network and a nonlinear support vector machine was used to classify subjects based on functional connectivity alone.

We achieved 80% cross-validated classification accuracy informed by a small set of discriminative edges. These edges connected a number of functional nodes in subcortical and cortical grey matter, and most were stronger in term neonates compared to those born preterm. Half of the discriminative edges connected one or more nodes within the basal ganglia.

These results demonstrate that functional connectivity in the preterm brain is significantly altered by term-equivalent age, confirming previous reports of altered connectivity between subcortical structures and higher-level association cortex following preterm birth.

## Highlights

• Robust classification of preterm and term-born neonates using functional connectivity patterns.
• Discriminative pattern of alterations in basal ganglia and frontal connections.
• Reflects system-wide disruption of subcortical–cortical connections following preterm birth.

## Introduction

The incidence of preterm birth is increasing and, with improved survival rates, is linked to a wide-ranging set of neurodevelopmental, cognitive and behavioural disorders (Delobel-Ayoub et al., 2009, Marlow et al., 2005, Moore et al., 2012). Identifying and understanding the mechanisms underpinning these disorders is paramount to the process of identifying possible therapeutic targets and developing early treatment strategies for this vulnerable population (Ment et al., 2009).

Magnetic resonance imaging (MRI) provides a non-invasive, *in vivo* strategy to study the developing brain. Using MRI, studies have found significant alterations to the size, complexity and structure of cerebral white and grey matter in preterm infants compared to their term-born counterparts (Boardman et al., 2006, Dubois et al., 2010, Inder et al., 2005, Kapellou et al., 2006). Alongside reduced cortical volume and complexity, the use of diffusion MRI has revealed significant differences in the microstructure throughout the white matter as early as term-equivalent age, even in the absence of major white matter pathologies (Anjari et al., 2007, Counsell et al., 2003, Counsell et al., 2006, Huppi et al., 1998). The deep grey matter structures (basal ganglia and thalami) in particular appear to be vulnerable following preterm birth: thalamic volume is significantly reduced in preterm infants, and linearly related to the degree of prematurity (Ball et al., 2012, Boardman et al., 2006). Thalamocortical connections are also disrupted by prematurity (Ball et al., 2013a), undermining the overall structural connectivity of the preterm brain (Ball et al., 2014). This evidence suggests that disruption of these systems may, in part, underlie subsequent adverse neurodevelopment in this population (Ball et al., 2015, Boardman et al., 2010, Fischi-Gómez et al., 2014, Volpe, 2009).

With such apparent structural disruptions, it would perhaps be expected that the functional state of the preterm brain would be substantially altered by term-equivalent age. Resting-state functional MRI (fMRI) can provide indirect evidence for intrinsic neural activity by exploiting the correlated interactions of spontaneous blood oxygen level–dependent (BOLD) signals across the brain (Biswal et al., 1995). Large-scale resting-state networks (RSN) have been identified in adult populations that correspond to activation patterns commonly derived from task-based fMRI (Smith et al., 2009). RSN are dominated by low-frequency BOLD fluctuations, have distinct spatial patterns and time courses and are present even during sleep and anaesthesia (Fukunaga et al., 2006, Vincent et al., 2007). Functional MRI is being increasingly used to examine brain development in the preterm period. Doria et al. (2010) demonstrated the presence, and striking similarity to adults, of RSN in the preterm brain by term-equivalent age. Notably, these networks were not significantly different from those found in healthy term-born infants. In contrast, using a seed-based approach, Smyser et al. (2010) observed a significant difference in thalamic-cortical connectivity in preterm infants, and more recently reported a reduction in the complexity and magnitude of interactions between RSN in the preterm brain (Smyser et al., 2014). In a detailed study of thalamocortical connectivity, Toulmin et al. (2015) also found significant reductions in connection strength between high-order cortical regions and specific thalamic nuclei in preterm infants. Other studies have reported an immature, or incomplete, repertoire of RSN in preterm infants present by term-equivalent age, although without a direct comparison between preterm and term-born populations (Fransson et al., 2007, Fransson et al., 2011, Gao et al., 2014, Lin et al., 2008).

In this study, we employ machine-learning techniques to model functional connectivity in the newborn brain. By combining high-dimensional independent component analysis (ICA) decomposition with multivariate machine-learning techniques, we test the hypothesis that preterm birth results in specific alterations to functional connectivity by term-equivalent age. We aim to use a data-driven approach to quantify functional connectivity in resting-state networks across the whole brain, characterising aspects of connectivity that best discriminate between preterm and term individuals.

## Methods

### Subjects

Ethical permission for this study was granted by the City and East London, and Hammersmith and Queen Charlotte's and Chelsea Hospital Research Ethics Committees. Written parental consent was obtained for each infant prior to imaging. Infants born before 37 weeks gestation and scanned at term-equivalent age (after 39 weeks postmenstrual age) between January 2008 and April 2013, with both resting state functional MRI and T2-weighted MRI available were considered for this study (*n* = 274). Exclusion criteria included: focal abnormality on MR imaging (including periventricular leukomalacia and haemorrhagic parenchymal infarction; *n* = 22), image acquisition artefacts precluding registration or segmentation of T2-weighted MRI (*n* = 3), non-motion-related fMRI image artefacts (*n* = 1) and excessive fMRI motion (*n* = 144; see Supplemental Materials for details).

In addition, images were acquired from 54 term-born control infants between November 2010 and February 2013. After quality inspection, as above, 28 datasets were excluded from further analysis due to excessive motion during fMRI acquisition.

The final study population consisted of 105 preterm-at-term infants (55 male) with a median gestational age (GA) at birth of 30^+ 1^ weeks (range: 23^+ 6^–34^+ 5^ weeks) and a median postmenstrual age (PMA) at scan of 42^+ 1^ weeks (range: 39^+ 0^–48^+ 0^ weeks), and 26 term-born infants (13 male) with a median GA of 39^+ 5^ weeks (range: 37^+ 1^–41^+ 6^ weeks) and a median PMA at scan of 43^+ 1^ weeks (range: 39^+ 5^–46^+ 2^ weeks).

### MRI acquisition

Each subject underwent T2-weighted MRI and resting-state fMRI acquisition. MRI was performed on a Philips 3-Tesla system (Philips Medical Systems, Best, The Netherlands) within the Neonatal Intensive Care Unit using an 8-channel phased array head coil.

T2-weighted fast-spin echo MRI was acquired using TR: 8670 ms; TE: 160 ms; flip angle: 90°; field-of-view: 220 mm^2^; matrix: 256 × 256 (voxel size: 0.86 × 0.86 × 2 mm) with 1 mm overlap. T2*-weighted functional MRI was acquired using an echo-planar imaging acquisition at TR: 1500 ms; TE: 45 ms; flip angle: 90°; field-of-view: 200 mm; matrix: 80 × 80 (voxel size: 2.5 × 2.5 × 4 mm), 256 volumes (total scan time = 6 min 24 s).

Sedation (25–50 mg/kg oral chloral hydrate) was administered to 126 infants prior to imaging (96.2%). Pulse oximetry, temperature and heart rate were monitored throughout and ear protection was used for each infant (President Putty, Coltene Whaledent, Mahwah, NJ; MiniMuffs, Natus Medical Inc., San Carlos, CA).

### Image processing

**Fig. 1:**
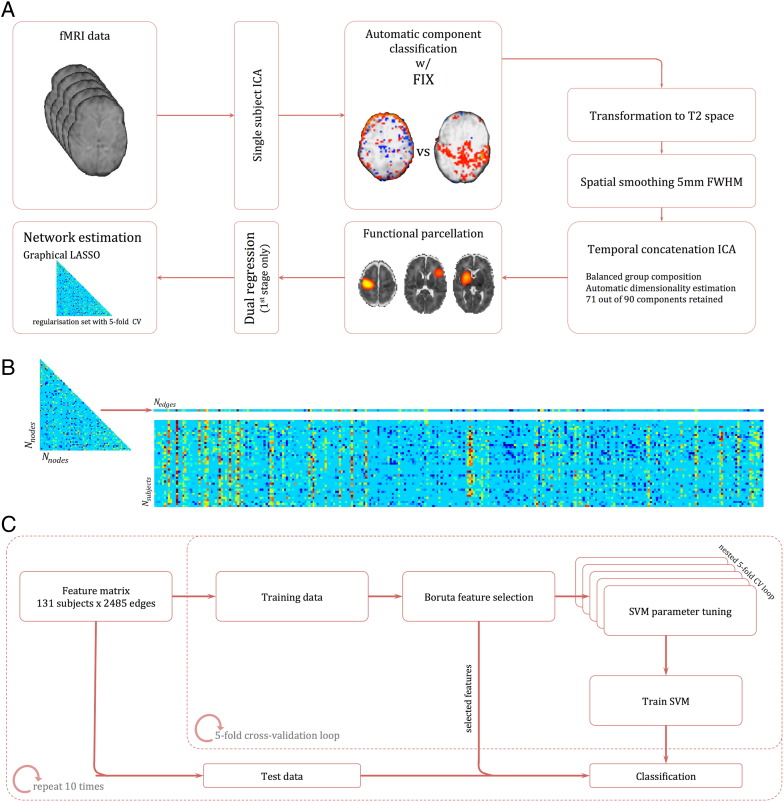
Processing pipeline. (A) Individual fMRI datasets were first denoised following single-subject ICA using FIX. Denoised data were then co-registered to corresponding anatomical T2 scans, smoothed and group ICA was performed in a balanced subset. ICA components were selected and spatially regressed onto individual datasets to extract time courses for each component—one per subject. Regularised partial correlation matrices were estimated for each subject. (B) Each connectivity matrix was reshaped to a vector of length *N*_edges_ and concatenated to form a feature matrix. (C) This matrix was repeatedly split into training and test data within a 5-fold cross-validation loop. Discriminative features were selected using a random forest–based, all-relevant selection procedure and passed to a nonlinear SVM for classification. The whole CV loop was repeated ten times.

The full processing pipeline is shown in Fig. 1. After motion assessment, all remaining datasets underwent single-subject ICA followed by FMRIB's FIX (Salimi-Khorshidi et al., 2014) for automatic de-noising and artefact removal prior to further analysis (Fig. 1A; see Supplemental Materials for details). Single-subject ICA was performed using MELODIC (Beckmann and Smith, 2004) on each dataset as follows: removal of the first 6 volumes to allow for T1 equilibration; motion correction with MCFLIRT; high-pass filter (125 s cutoff, 0.008 Hz); and automatic dimensionality estimation. To preserve high-frequency spatial and temporal signal prior to FIX processing, no slice timing correction or spatial smoothing was applied. Empirical evidence suggests that the application of slice-timing correction does not affect resting state functional connectivity metrics (Wu et al., 2011). The standard FIX processing steps were modified to allow for standard-space masking using a population-specific neonatal template with tissue priors rather than the adult MNI152 atlas (Serag et al., 2012).

**Fig. S1:**
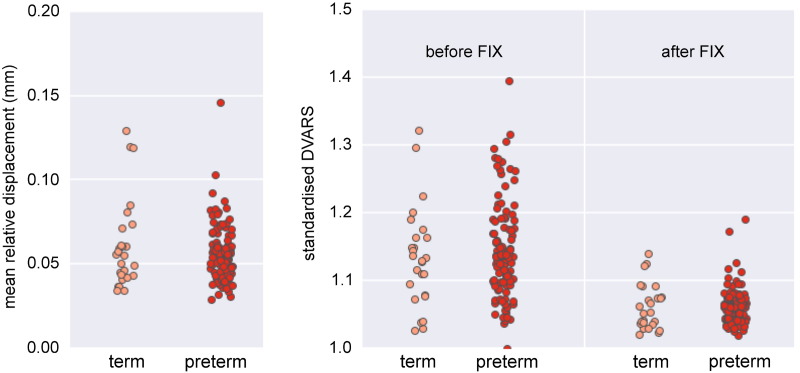
Motion parameters in each group. Mean relative (frame-to-frame) displacement (left panel) and standardised DVARS, before and after FIX processing (right panel) are shown for each group.

After automatic component classification, the unique variance of each noise component was regressed out of the data alongside the full variance of the motion parameters and derivatives (Griffanti et al., 2014, Satterthwaite et al., 2013). Standardised DVARS—the spatial standard deviation of the temporal derivative of the data, normalized by the temporal standard deviation and temporal autocorrelation (Power et al., 2012) (see also T. Nichols' discussion of DVARS; Nichols, 2013) was calculated before and after FIX clean-up for all subjects. In this case, a value closer to 1 indicates ‘cleaner’ fMRI data. DVARS was significantly reduced after FIX clean-up in both preterm (*t* = 13.6, *p* < 0.001) and term cohorts (*t* = 6.8, *p* < 0.001). Mean relative, frame-to-frame displacement (MRD) and DVARS before and after FIX clean-up for both groups are shown in Fig. S1. MRD and DVARS (before or after FIX) did not differ significantly between term and preterm cohorts (*t* = − 1.1, *p* = 0.27; *t* = 0.63, *p* = 0.53; and *t* = − 0.07, *p* = 0.95, respectively).

### Group ICA

**Fig. S2:**
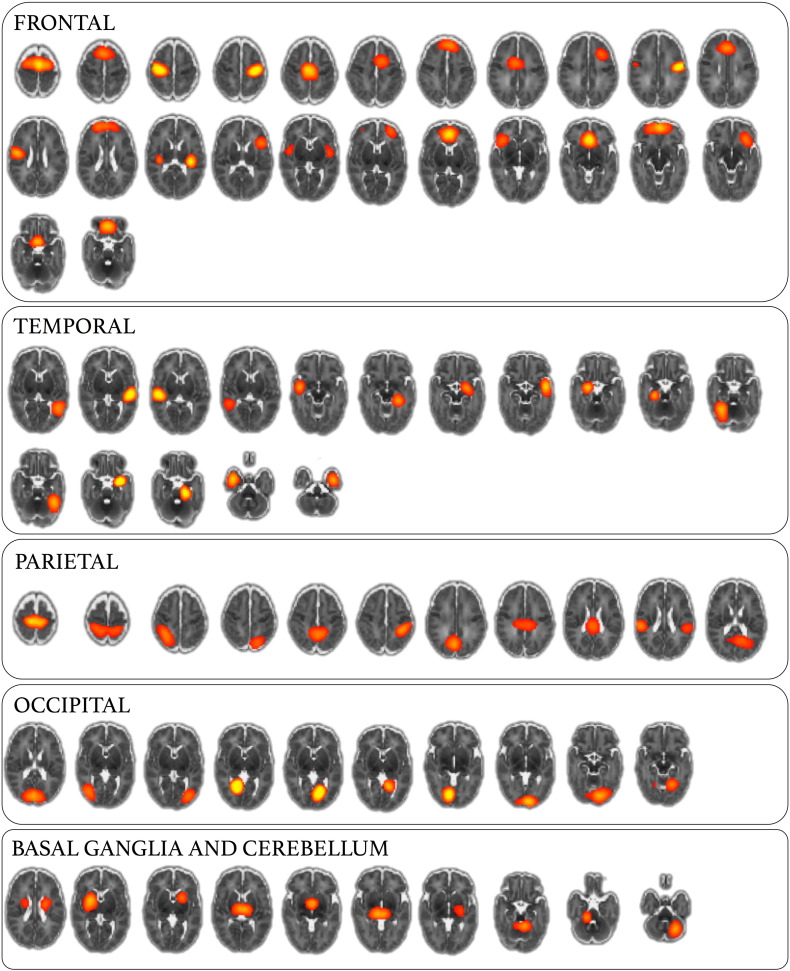
Functional parcellation via higher-order ICA. A total of 71 ICA components used for functional parcellation are shown at *Z* = 10, ordered according to cortical lobe/region.

**Fig. S3:**
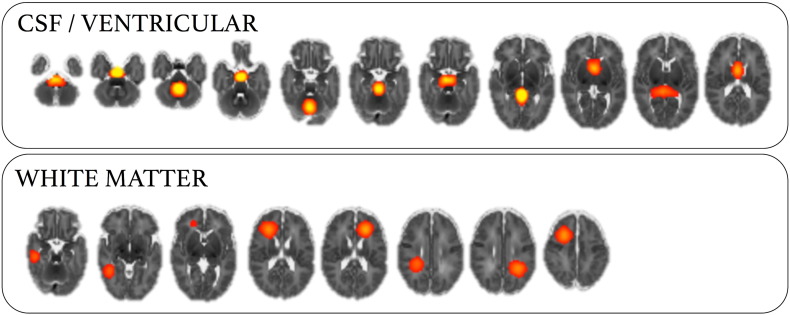
ICA components identified as noise or artefact. A total of 19 ICA components were identified as probable artefacts with non-neuronal source based on manual inspection of the spatial distribution (centred in CSF or WM) and frequency power spectrum (shown at *Z* = 10).

In order to examine whole-brain functional connectivity, it is first necessary to parcellate the brain into a set of functional regions, or nodes (Smith et al., 2013, Varoquaux and Craddock, 2013). Ideally, nodes should be functionally homogeneous and well-matched across subjects; simulations show that poor node definition can lead to errors in network estimation (Smith et al., 2011). Here, we construct a functional parcellation by performing group ICA (Beckmann and Smith, 2004, Kiviniemi et al., 2009, Smith et al., 2009). ICA-based parcellation tends to be relatively robust to group composition (Varoquaux et al., 2010a, Varoquaux et al., 2010b); however, due to the unbalanced nature of the dataset, we attempted to limit any possible bias in the parcellation toward the larger, preterm cohort by choosing a subset of 26 randomly selected preterm subjects to create a balanced functional parcellation. Functional MRI data from all term subjects and the preterm subset were registered to a population-specific T2-weighted template, resampled to 2 mm isotropic voxels and smoothed with a Gaussian kernel of 5 mm FWHM. Temporal concatenation ICA was performed with MELODIC, the number of components was determined automatically. Of the resulting 90 components, 19 were discarded after visual inspection of both the component's spatial distribution and frequency power spectrum. Components were rejected if the spatial maps predominantly contained ventricular CSF or white matter regions and/or if the content of the power spectra was contaminated by high-frequency signal. The remaining set of 71 components (nodes) were used to generate subject-specific time series—one per component—using dual regression (Filippini et al., 2009). Only the first stage of dual regression was performed, using the (unthresholded) group level component maps as spatial regressors to produce a set of subject-specific time courses for each component to enter into network modeling. Full functional parcellation is shown in Fig. S2 (components rejected as artefacts are shown in Fig S3). This approach has the benefit of capturing subject-specific functional information, without the *a priori* definition of functional regions using anatomical atlases, or the use of landmark-based seeds.

### Network analysis

Subject-specific functional connectivity networks were estimated using an L_1_-regularised graphical lasso model (Friedman et al., 2008) implemented in Scikit-learn (0.15.2) (Pedregosa et al., 2011). A penalised optimisation method, the graphical lasso imposes a penalty on the off-diagonal entries of the inverse covariance matrix, shrinking estimated values of pairwise partial correlations, forcing small or noisy values to zero and resulting in a sparse matrix of direct connections (Friedman et al., 2008). This method is beneficial when the number of samples is relatively small in comparison to the number of observations (nodes) has been shown to most effectively recover connectivity structure in real and simulated fMRI data (Smith et al., 2011, Varoquaux and Craddock, 2013). For each subject, the L_1_-regularisation penalty was set using 5-fold cross-validation, minimising the generalisation error between training and test data along a descending path of iteratively refined regularisation terms. The final sparse solution was estimated for each subject and the resulting precision matrices were converted to partial correlations. Finally, Fisher's *r*-to-*z* transformation was applied to each partial correlation matrix. For comparison, unregularised partial correlation matrices and full correlation matrices were also calculated for each subject.

### Feature selection and classification

The subject's *N*_node_ × *N*_node_ connectivity matrices were reordered and concatenated to give an *N*_subject_ × *N*_edge_ matrix (Fig. 1B), where each element represents connectivity strength (given by the *z*-transformed, regularised partial correlation) between two nodes for a given subject. Classification accuracy and generalisation error was estimated using 10-repeat, 5-fold cross-validation (CV; Fig. 1C). Feature selection was performed using the Boruta algorithm (implemented in Python: bitbucket.org/danielhomola/boruta_py), an all-relevant feature selection method based on random forests (Kursa and Rudnicki, 2010). Briefly, the feature set is copied and shuffled to create a set of ‘shadow’ features. Random forest classification is performed (500 pruned decision trees with a maximum depth of 5) and the Gini importance of both features and shadow features is calculated. After each iteration, features with importance significantly greater than the 95th percentile of shadow features (assessed using a binomial distribution) were selected for further analysis and removed (along with the corresponding shadow feature) from the next iteration. A two-step process is used to account for multiple comparisons: at each iteration, FDR correction (*q* = 0.05) is applied over all active features in the random forest and, once selected, a Bonferroni correction (*p* < 0.05) is applied over all iterations for selected features (such that the selection criterion becomes more stringent with increasing number of iterations/repeated tests). In total, 250 random forest iterations were performed, ultimately leaving a set of features deemed relevant to the classification problem.

For each training dataset, the reduced set of discriminative features was passed to a support vector machine with radial basis function kernel (SVM-RBF; Scikit-learn 0.15.2), where hyperparameters *γ* and *C* were tuned using a nested, stratified 5-fold cross-validation to maximise the balanced accuracy (mean of specificity and sensitivity). Due to the unbalanced nature of the dataset, a proportional sample weight was applied in each case to the smaller class in each fold. Finally, the trained SVM was used to classify the left-out test data. This was repeated for all five folds, and the whole CV process was repeated ten times (Fig. 1C).

## Results

### Classification

**Fig. 2:**
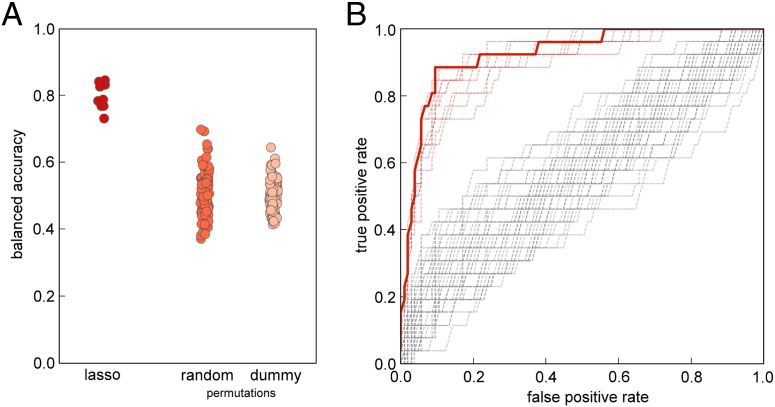
Classification accuracy and feature selection. (A) Balanced classification accuracy estimated over ten 5-fold CV repeats, compared to 100 (out of 1000) random permutations and dummy classifications based on the empirical distribution of class labels. (B) Classification ROC curve in red (showing the mean class probability averaged over all ten repeats), with individual CV repeats shown as red dashed lines. ROC curves from 100 (out of 1000) random permutations are shown in grey.

Group status of each subject was predicted with a mean (± SD) balanced accuracy of 80.2% (± 3.8%) (Fig. 2A). The area under the ROC curve was 0.92 (± 0.016) (Fig. 2B). Classification accuracy was higher when connections were estimated with the graphical lasso model, compared to both unregularised partial correlation (mean balanced accuracy: 61.1 ± 3.0%; paired *t*-test: *p* < 0.001) and full correlation (75.5 ± 2.2%; *p* < 0.05).

**Fig. S4:**
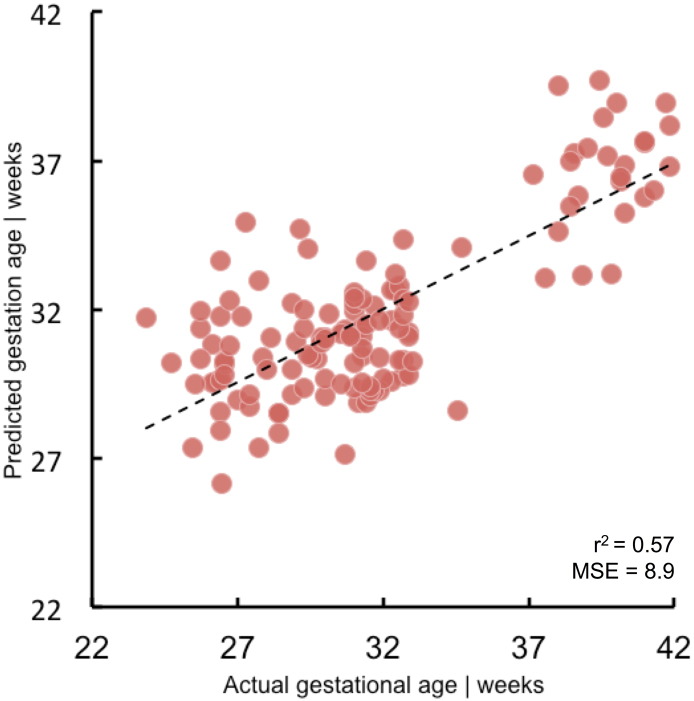
Predicting gestational age using SVM regression. Features selected in the classification model were also used to predict GA at birth using SVM regression (with hyperparameters tuned to minimise MSE). The relationship between predicted GA (averaged over 10 CV repeats) and the true GA of each infant is shown.

We compared classification accuracy to two null models (Fig. 2A). Initially, by performing a dummy classification: randomly labelling the test data with a probability based on the empirical distribution of classes within the training data. This process was repeated 1000 times and resulted in a balanced accuracy of 50.0 ± 4.4%. Secondly, we performed 1000 random permutations where group labels were permuted prior to feature selection and classifier training (balanced accuracy: 50.2 ± 5.8%). Classification accuracies of the first 100 permutations of each null model are shown alongside all ten CV repeats of the full model in Fig. 2A. The full model significantly outperformed both null models (*p* < 0.001). We also performed a regression analysis, using the selected edges to predict the gestational age at birth of each infant. The relationship between predicted GA (averaged over ten CV folds) and true GA is shown in Fig S4, the model was able to predict GA across the whole age range (*r*^2^ = 0.57, MSE = 8.9).

In order to determine if differences in motion between preterm and term groups influenced classification accuracy, mean relative displacement (MRD) was added as an extra feature to each training set. Additionally, classification was performed using just MRD (i.e., no connectivity features). After cross-validation, the addition of MRD to the connectivity did not alter classification accuracy (80.2 ± 3.9%). Classification with MRD alone resulted in a balanced accuracy of 50.6 ± 3.4%.

Classification error was significantly higher in the term cohort (mean over ten CV repeats: 33.1%) compared to the preterm cohort (mean: 5.3%). In both groups, we found that mean misclassification rates did not correlate with intracranial volume size, age at scan, or mean MRD (*p* > 0.05 for all). A tendency towards higher misclassification in gestationally younger term control infants was observed (*r* = − 0.29, *p* = 0.15) although this was not the case in the preterm cohort. These basic measures suggest that misclassification in either group was not driven by gross anatomical differences, or by different motion profiles in the misclassified cases. To investigate the effects of sample size on misclassification, we randomly undersampled the preterm cohort to create a balanced dataset of equal sample size and repeated the classification. In the balanced dataset, classification accuracy was reduced (mean ± SD: 78.9 ± 4.5%) and misclassification rates were similar between term and preterm cohorts (18.1 ± 6.8% and 24.0 ± 4.6%, respectively). With a smaller training sample, classification error increased significantly in the preterm cohort, suggesting that the higher misclassification rate observed in the term cohort in the full model is likely to be an effect of the unbalanced dataset rather than a latent confounding variable.

### Important features

**Fig. 3:**
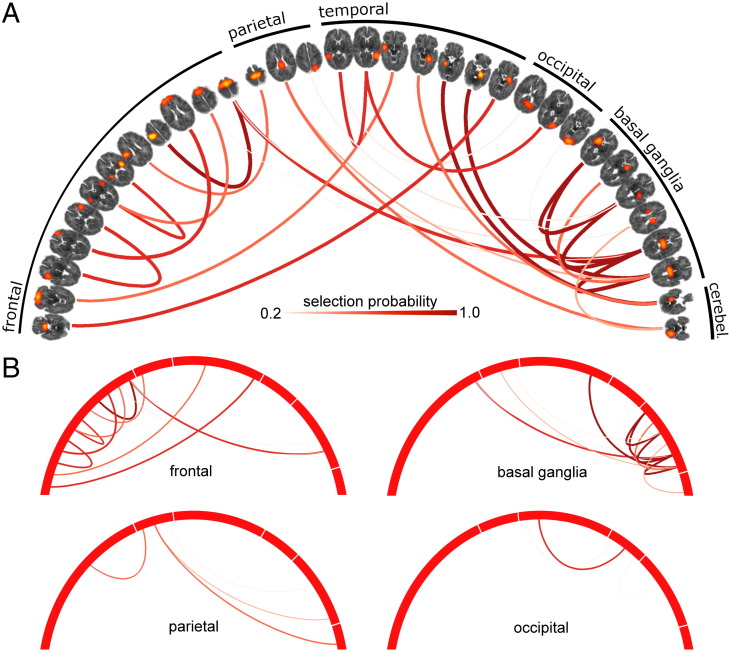
Discriminative edges for the classification of preterm and term infants. (A) Discriminative edges selected by the classification model, visualised with Circos (Krzywinski et al., 2009). Connected nodes are shown ordered according to cortical lobe/region and are thresholded at *Z* = 10. Edge colour and width reflects selection probability. (B) Edges are clustered according to region, such that all edges that connect to at least one node in each region are shown.

For each CV repeat, feature (network edge) selection probability was averaged across all five training folds and compared to the corresponding selection probability from 1000 random permutations of the full model (where group labels were permuted prior to feature selection). Edges that were selected significantly more frequently than in the permuted models (*p* < 0.01) were identified and a subset of 27 discriminative edges that were consistently selected over all ten CV repeats is shown in Fig. 3.

In total, 33 functional nodes were connected by the set of discriminative edges (Fig. 3A). Connected functional regions included medial and lateral frontal cortex, anterior and posterior insula, and primary and supplementary motor areas in the frontal lobe; primary sensory and posterior cingulate cortex in the parietal lobe; medial temporal structures including hippocampi, and the tempo-parietal junction; primary visual cortex; cerebellum; and in the basal ganglia, bilateral thalamic and putamen nodes. The majority of edges (16/27) connected nodes in different regions/lobes.

Of the 27 discriminative edges: 13 connected to at least one basal ganglia node, 11 connected to at least one frontal node, 4 edges connected at least one parietal node, 4 connected to cerebellar nodes, and 3 connected occipital nodes (Fig. 3B; cerebellar connections not shown separately). After randomly selecting 27/2485 edges over 1000 permutations, we found that only the number of basal ganglia connections exceeded the expected number of connections given the number of nodes in each region (mean number of basal ganglia edges from permutation models ± SD: 6.3 ± 2.18, *p* < 0.003).

**Fig. 4:**
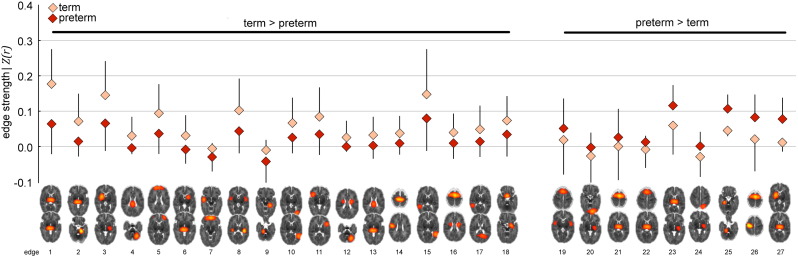
Discriminative edge strength in preterm and term infants. Edge strengths of discriminative edges for each group, ordered by effect size from left to right (see also Table 1).

The strength of each discriminative edge, as measured by its *z*-transformed partial correlation, is shown in Fig. 4 for both groups, and mean edge strengths are shown in Table 1. Eighteen (66%) of the discriminative edges were, on average, stronger in the term group compared to the preterm group (arranged from left to right by effect size in Fig. 4). Large strength differences (in both directions) were observed in basal ganglia connections (e.g., edges 1, 2, 3, 23 and 27) and frontal connections (e.g., edges 5, 8, 11 and 26). Absolute effect size (Cohen's *d*) of the difference in edge strengths also significantly correlated with the mean edge selection probability (*r* = 0.57, *p* < 0.01; Table 1).

## Discussion

By applying machine-learning methods to the whole-brain neonatal functional connectome, we have been able to discriminate between functional connectivity data acquired from preterm infants at term-equivalent age and healthy term-born controls with 80% accuracy. The functional connectivity of the preterm brain was characterised by alterations to a complex set of interactions between functional nodes in both subcortical and cortical grey matter. The identification of a set of connections predominantly between functional nodes within the basal ganglia and nodes in the frontal cortex confirms previous findings that the subcortical grey matter is particularly vulnerable to injury or maldevelopment after preterm birth (Ball et al., 2012, Boardman et al., 2010, Smyser et al., 2010), and that prematurity adversely affects subcortical–cortical systems as a whole (Ball et al., 2013a, Fischi-Gómez et al., 2014, Toulmin et al., 2015).

Previous reports have demonstrated the relatively advanced organisational state of spontaneous BOLD fluctuations in the preterm brain, with many RSN present in adults evident in preterm infants by term-equivalent age and present in healthy term-born neonates (Doria et al., 2010, Fransson et al., 2009). Evidence suggests an asynchronous development of networks in accordance with a functional hierarchy: early maturing primary sensory areas—visual and auditory cortices—preceding the emergence of the default mode and attentional networks in frontal and parietal regions (Fransson et al., 2011), in line with microstructural development of the cortex during the same period (Ball et al., 2013b). By 18 months of age, RSN are apparently indistinguishable between preterm and term-born infants, but connections between RSN are significantly stronger in those born at term (Damaraju et al., 2010).

The early establishment of subcortical RSN activity was noted by Doria et al. (2010) and Fransson et al. (2009) and, using a seed-based approach, Smyser et al. (2010) found that connectivity between the thalamus and the motor cortex appeared to be reduced following preterm birth. Toulmin et al. (2015) recently performed a comprehensive examination of thalamocortical connectivity in preterm infants and demonstrated that the functional organisation of the thalamus reveals a concise description of cortical connectivity at term-equivalent age, closely resembling that reported in adults (Zhang et al., 2008). Additionally, specific deficits in thalamic connections to superior frontal and anterior cingulate cortices were observed when compared to term-born controls. (Toulmin et al., 2015)

We confirm these findings, demonstrating that disruptions to cortical connectivity in the preterm brain also involve several basal ganglia nodes and connections between higher-level cortical regions. In general, absolute connectivity strength of discriminative edges was reduced in the preterm cohort. This suggests that events occurring during the preterm period impact negatively on the development of functional connectivity during a critical period of vulnerability, reflecting the impact on underlying structural connectivity previously reported in this population (Ball et al., 2013a, Ball et al., 2014).

During development, spontaneous neuronal activity is feasible in the cortical subplate from mid-gestation, as pioneering cortico-cortical and thalamocortical fibres begin to synapse and form transient circuitry (Kostovic and Judas, 2006, Moore et al., 2011). This begins to give way to layer-specific, sensory-driven cortical circuitry during the third trimester, as evidenced by the increasing synchronicity of spontaneous transient activity and establishment of oscillatory EEG activity during the preterm period (Vanhatalo and Kaila, 2006). The subplate itself is crucial to the development of cortical lamination and circuitry, and is specifically vulnerable to the rigour of preterm birth (Allendoerfer and Shatz, 1994, Ghosh et al., 1990, McQuillen et al., 2003). Indeed, hypoxia-mediated inflammation following preterm birth can result in damage to specific cell populations that support long-range cerebral connectivity (Back et al., 2001, McQuillen et al., 2003, Segovia et al., 2008). This may lead to delay or damage to the formation of emerging cortical circuits, resulting in the aberrant long-distance cortical and subcortical connectivity observed here. Structural MRI has revealed decreased volume specific to the basal ganglia in preterm infants, compared to term-born controls (Boardman et al., 2006), and thalamic and cortical growth is dependent on the degree of prematurity (Ball et al., 2012), In addition, cortical volume, deep grey matter growth, and microstructural measures of thalamocortical connectivity at term-equivalent age all correlate strongly with neurodevelopmental outcome in early childhood in preterm populations (Ball et al., 2015, Rathbone et al., 2011, Young et al., 2015). This suggests that the discriminative pattern of functional connectivity described here reflects the complex patterning of structural and microstructural disturbances previously reported in preterm neonates, reinforcing the hypothesis that preterm brain injury involves a complex interaction of processes across tissue compartments and brain regions (Volpe, 2009).

In order to discriminate between preterm and term connectivity data, we combined multivariate feature selection and classification techniques, with sparse network estimation and functional parcellation of the neonatal brain. In network analysis, how nodes are defined can significantly affect the topology of the resulting network, and inappropriate node choice—such as a coarse atlas with boundary errors between functionally distinct regions together—can result in significant errors in network estimation (Smith et al., 2011). Defining network nodes using (high-order) ICA-based techniques allows the capture of population-specific functional topology without an *a priori* definition of regions-of-interest (Kiviniemi et al., 2009). This is particularly appropriate for developmental populations when population-specific anatomical atlases, or well-characterised functional parcellations, may not be available. Defining an optimal number of nodes is difficult; studies have shown that between-group differences can be mediated by ICA model order (Abou Elseoud et al., 2011), and lower-order models (10–20 components) return large, spatially fragmented nodes, hindering the interpretation of connections between them. We defined 71 functional nodes in our population, this is in line with previous studies in developmental populations (Damaraju et al., 2014) and offered good cerebral coverage coupled with anatomically distinct nodes (Fig. S2). Network connectivity was estimated using a regularised graphical lasso model. This method has been shown to be very effective in recovering network structure in both simulated and real fMRI datasets (Smith et al., 2011). The estimation of partial correlations via a precision matrix can result in noisy estimates, particularly when the number of observations (fMRI volumes) does not greatly outnumber the number of features (functional nodes). This process is stabilised with the introduction of regularisation (Friedman et al., 2008). Here, we show that graphical lasso resulted in the highest classification accuracy between term and preterm datasets. We believe this supports the hypothesis that regularised partial correlation matrices provide a better estimate of the true macroscopic functional connectome than full correlation (Smith et al., 2011, Varoquaux and Craddock, 2013). A limitation of this approach is that regularisation assumes a level of sparsity in the connectivity matrix that is not necessarily shared across subjects. We estimated sparsity on an individual level using an internal CV loop, resulting in a slightly different network topology in each subject and some discriminative edges that were present in one group only (Fig. 4). An alternative would be to employ a prior over the whole group to constrain network topology while allowing edge weights to vary (Varoquaux et al., 2010a, Varoquaux et al., 2010b). While an attractive proposition, the extent to which network topology should be constrained across different developmental or pathological cohorts is unclear.

Due to the multivariate nature of the analysis, we note that care should be taken in the interpretation of the strength of individual discriminative edges, as it is the pattern of connectivity across all discriminative edges that provides the classification of preterm or term data. Similarly, it is also likely that these connections are not the only ones that differ, on average, between the two groups (Smyser et al., 2014). Classification error was significantly higher in the term group than in the preterm group. We believe classification accuracy in the term infants was limited by the relatively small sample size. The recruitment of healthy, term-born control infants is difficult and limited by ethical concerns surrounding experimental procedures such as sedation in healthy neonatal populations. These difficulties often lead to class imbalances in larger, neonatal MRI studies of prematurity where only a limited number of control infants are available for study (Anblagan et al., 2015, Ceschin et al., 2015, Padilla et al., 2014, Pannek et al., 2013, Toulmin et al., 2015). Although we used cost-based weighting in order to mitigate misclassification error with some success, other approaches, including undersampling of the majority class and replication or simulation of minority cases (e.g., SMOTE) (Chawla et al., 2002), may also provide avenues for further research in this field. Additionally, although MRI images were assessed for any pathology that may lead to abnormal neurodevelopment, behavioural follow-up testing of the control cohort is currently ongoing so we are unable to confirm at this time that all control infants are within the normal developmental range.

Finally, motion artefacts in fMRI are well-known to be problematic for connectivity estimation, particularly in developmental cohorts (Power et al., 2012, Van Dijk et al., 2012). In this study, 96% of the infants were sedated prior to scanning, and stringent motion thresholds were employed. All infants were sedated with similar doses (25–50 mg/kg) and sedation was not re-administered to any infants during the scan. Importantly, the order in which scan sequences were acquired was also the same for all individuals, such that the level of sedation at the time of fMRI acquisition would be expected to be similar across subjects. We did not anticipate a confounding influence of sedation in the present study and repeating the classification after removing the five non-sedated preterm infants did not alter the model accuracy (balanced accuracy: 84.0%). We have also previously shown that both ICA-based network topology and global CBF is robust to sedation state in neonates (Arichi et al., 2012, Doria et al., 2010). To account for motion effects, we applied an ICA-based classification algorithm designed to denoise fMRI datasets (Salimi-Khorshidi et al., 2014), and regressed out motion parameters (and a full set of temporal derivatives) from the data prior to network estimation. The amount of relative, frame-to-frame motion (MRD) did not significantly differ between preterm and term infants nor did DVARS after FIX processing, suggesting that the groups were well matched for motion. Finally, we used MRD alone to try and classify preterm and term infants. This only achieved a mean balanced accuracy of 50.6%, suggesting that our classification was driven by a true measure of connection strength rather than motion effects.

## Conclusions

While functional connectivity networks are well established early in development in both preterm and term-born neonates, there remains a significant difference between the two populations. Here, we demonstrate that functional connections of the basal ganglia and higher-level frontal regions are significantly altered in preterm infants by term-equivalent age. This mirrors previous findings of reduced structural connectivity in the developing preterm brain and indicates a system-wide disruption of subcortical–cortical connections following preterm birth. Machine-learning methods open avenues to explore other influences on the developing connectome in this population, for example, genetic susceptibilities or clinical environment, and to further improve our understanding of the preterm brain.

The following are the supplementary data related to this article.

Supplementary material.

## Figures and Tables

**Table 1:** Mean strengths and selection probabilities for discriminative edges.

	Mean edge strength			
Edge⁎	Term	Preterm	Cohen's *d*	Selection probability
1	0.177	0.064	1.23	1.00
2	0.072	0.015	0.91	1.00
3	0.144	0.068	0.86	1.00
4	0.031	− 0.002	0.84	0.76
5	0.093	0.037	0.79	0.94
6	0.032	− 0.007	0.77	0.74
7	− 0.005	− 0.028	0.74	0.74
8	0.102	0.045	0.73	0.84
9	− 0.009	− 0.042	0.71	0.78
10	0.067	0.026	0.68	0.82
11	0.084	0.036	0.66	0.90
12	0.026	0.002	0.66	0.56
13	0.032	0.004	0.63	0.44
14	0.037	0.011	0.62	0.68
15	0.148	0.080	0.61	0.84
16	0.039	0.010	0.59	0.30
17	0.048	0.015	0.58	0.38
18	0.074	0.036	0.56	0.86
19	0.019	0.052	− 0.54	0.72
20	− 0.023	− 0.003	− 0.60	0.34
21	0.003	0.027	− 0.64	0.88
22	− 0.006	0.014	− 0.66	0.26
23	0.061	0.117	− 0.68	1.00
24	− 0.027	0.001	− 0.70	0.36
25	0.046	0.106	− 0.83	1.00
26	0.021	0.083	− 0.92	1.00
27	0.014	0.079	− 0.99	1.00

⁎ Corresponding in order to Fig. 4.

## References

[bb0005] Abou Elseoud A., Littow H., Remes J., Starck T., Nikkinen J., Nissilä J., Timonen M., Tervonen O., Kiviniemi V. (2011). Group-ICA model order highlights patterns of functional brain connectivity. Front. Syst. Neurosci..

[bb0010] Allendoerfer K.L., Shatz C.J. (1994). The subplate, a transient neocortical structure: its role in the development of connections between thalamus and cortex. Annu. Rev. Neurosci..

[bb0015] Anblagan D., Bastin M.E., Sparrow S., Piyasena C., Pataky R., Moore E.J., Serag A., Wilkinson A.G., Clayden J.D., Semple S.I., Boardman J.P. (2015). Tract shape modeling detects changes associated with preterm birth and neuroprotective treatment effects. NeuroImage Clin..

[bb0020] Anjari M., Srinivasan L., Allsop J.M., Hajnal J.V., Rutherford M.A., Edwards A.D., Counsell S.J. (2007). Diffusion tensor imaging with tract-based spatial statistics reveals local white matter abnormalities in preterm infants. NeuroImage.

[bb0025] Arichi T., Fagiolo G., Varela M., Melendez-Calderon A., Allievi A., Merchant N., Tusor N., Counsell S.J., Burdet E., Beckmann C.F., Edwards A.D. (2012). Development of BOLD signal hemodynamic responses in the human brain. NeuroImage.

[bb0030] Back S.A., Luo N.L., Borenstein N.S., Levine J.M., Volpe J.J., Kinney H.C. (2001). Late oligodendrocyte progenitors coincide with the developmental window of vulnerability for human perinatal white matter injury. J. Neurosci. Off. J. Soc. Neurosci..

[bb0045] Ball G., Boardman J.P., Rueckert D., Aljabar P., Arichi T., Merchant N., Gousias I.S., Edwards A.D., Counsell S.J. (2012). The effect of preterm birth on thalamic and cortical development. Cereb. Cortex.

[bb0040] Ball G., Boardman J.P., Aljabar P., Pandit A., Arichi T., Merchant N., Rueckert D., Edwards A.D., Counsell S.J. (2013). The influence of preterm birth on the developing thalamocortical connectome. Cortex J. Devoted Study Nerv. Syst. Behav..

[bb0055] Ball G., Srinivasan L., Aljabar P., Counsell S.J., Durighel G., Hajnal J.V., Rutherford M.A., Edwards A.D. (2013). Development of cortical microstructure in the preterm human brain. Proc. Natl. Acad. Sci. U. S. A..

[bb0035] Ball G., Aljabar P., Zebari S., Tusor N., Arichi T., Merchant N., Robinson E.C., Ogundipe E., Rueckert D., Edwards A.D., Counsell S.J. (2014). Rich-club organization of the newborn human brain. Proc. Natl. Acad. Sci..

[bb0050] Ball G., Pazderova L., Chew A., Tusor N., Merchant N., Arichi T., Allsop J.M., Cowan F.M., Edwards A.D., Counsell S.J. (2015). Thalamocortical connectivity predicts cognition in children born preterm. Cereb. Cortex.

[bb0060] Beckmann C.F., Smith S.M. (2004). Probabilistic independent component analysis for functional magnetic resonance imaging. IEEE Trans. Med. Imaging.

[bb0065] Biswal B., Yetkin F.Z., Haughton V.M., Hyde J.S. (1995). Functional connectivity in the motor cortex of resting human brain using echo-planar MRI. Magn. Reson. Med. Off. J. Soc. Magn. Reson. Med. Soc. Magn. Reson. Med..

[bb0070] Boardman J.P., Counsell S.J., Rueckert D., Kapellou O., Bhatia K.K., Aljabar P., Hajnal J., Allsop J.M., Rutherford M.A., Edwards A.D. (2006). Abnormal deep grey matter development following preterm birth detected using deformation-based morphometry. NeuroImage.

[bb0075] Boardman J.P., Craven C., Valappil S., Counsell S.J., Dyet L.E., Rueckert D., Aljabar P., Rutherford M.A., Chew A.T., Allsop J.M., Cowan F., Edwards A.D. (2010). A common neonatal image phenotype predicts adverse neurodevelopmental outcome in children born preterm. NeuroImage.

[bb0080] Ceschin R., Wisnowski J.L., Paquette L.B., Nelson M.D., Blüml S., Panigrahy A. (2015). Developmental synergy between thalamic structure and interhemispheric connectivity in the visual system of preterm infants. NeuroImage Clin..

[bb0085] Chawla N.V., Bowyer K.W., Hall L.O., Kegelmeyer W.P. (2002). SMOTE: synthetic minority over-sampling technique. J. Artif. Intell. Res..

[bb0090] Counsell S.J., Allsop J.M., Harrison M.C., Larkman D.J., Kennea N.L., Kapellou O., Cowan F.M., Hajnal J.V., Edwards A.D., Rutherford M.A. (2003). Diffusion-weighted imaging of the brain in preterm infants with focal and diffuse white matter abnormality. Pediatrics.

[bb0095] Counsell S.J., Shen Y., Boardman J.P., Larkman D.J., Kapellou O., Ward P., Allsop J.M., Cowan F.M., Hajnal J.V., Edwards A.D., Rutherford M.A. (2006). Axial and radial diffusivity in preterm infants who have diffuse white matter changes on magnetic resonance imaging at term-equivalent age. Pediatrics.

[bb0105] Damaraju E., Phillips J., Lowe J.R., Ohls R., Calhoun V.D., Caprihan A. (2010). Resting-state functional connectivity differences in premature children. Front. Syst. Neurosci..

[bb0100] Damaraju E., Caprihan A., Lowe J.R., Allen E.A., Calhoun V.D., Phillips J.P. (2014). Functional connectivity in the developing brain: a longitudinal study from 4 to 9 months of age. NeuroImage.

[bb0110] Delobel-Ayoub M., Arnaud C., White-Koning M., Casper C., Pierrat V., Garel M., Burguet A., Roze J.C., Matis J., Picaud J.C., Kaminski M., Larroque B., EPIPAGE Study Group (2009). Behavioral problems and cognitive performance at 5 years of age after very preterm birth: the EPIPAGE Study. Pediatrics.

[bb0115] Doria V., Beckmann C.F., Arichi T., Merchant N., Groppo M., Turkheimer F.E., Counsell S.J., Murgasova M., Aljabar P., Nunes R.G., Larkman D.J., Rees G., Edwards A.D. (2010). Emergence of resting state networks in the preterm human brain. Proc. Natl. Acad. Sci. U. S. A..

[bb0120] Dubois J., Benders M., Lazeyras F., Borradori-Tolsa C., Leuchter R.H.-V., Mangin J.F., Hüppi P.S. (2010). Structural asymmetries of perisylvian regions in the preterm newborn. NeuroImage.

[bb0125] Filippini N., MacIntosh B.J., Hough M.G., Goodwin G.M., Frisoni G.B., Smith S.M., Matthews P.M., Beckmann C.F., Mackay C.E. (2009). Distinct patterns of brain activity in young carriers of the APOE-epsilon4 allele. Proc. Natl. Acad. Sci. U. S. A..

[bb0130] Fischi-Gómez E., Vasung L., Meskaldji D.-E., Lazeyras F., Borradori-Tolsa C., Hagmann P., Barisnikov K., Thiran J.-P., Hüppi P.S. (2014). Structural brain connectivity in school-age preterm infants provides evidence for impaired networks relevant for higher order cognitive skills and social cognition. Cereb. Cortex.

[bb0145] Fransson P., Skiold B., Horsch S., Nordell A., Blennow M., Lagercrantz H., Aden U. (2007). Resting-state networks in the infant brain. Proc. Natl. Acad. Sci. U. S. A..

[bb0140] Fransson P., Skiöld B., Engström M., Hallberg B., Mosskin M., Åden U., Lagercrantz H., Blennow M. (2009). Spontaneous brain activity in the newborn brain during natural sleep—an fMRI study in infants born at full term. Pediatr. Res..

[bb0135] Fransson P., Åden U., Blennow M., Lagercrantz H. (2011). The functional architecture of the infant brain as revealed by resting-state fMRI. Cereb. Cortex.

[bb0150] Friedman J., Hastie T., Tibshirani R. (2008). Sparse inverse covariance estimation with the graphical lasso. Biostatistics.

[bb0155] Fukunaga M., Horovitz S.G., van Gelderen P., de Zwart J.A., Jansma J.M., Ikonomidou V.N., Chu R., Deckers R.H.R., Leopold D.A., Duyn J.H. (2006). Large-amplitude, spatially correlated fluctuations in BOLD fMRI signals during extended rest and early sleep stages. Magn. Reson. Imaging.

[bb0160] Gao W., Alcauter S., Smith J.K., Gilmore J.H., Lin W. (2014). Development of human brain cortical network architecture during infancy. Brain Struct. Funct..

[bb0165] Ghosh A., Antonini A., McConnell S.K., Shatz C.J. (1990). Requirement for subplate neurons in the formation of thalamocortical connections. Nature.

[bb0170] Griffanti L., Salimi-Khorshidi G., Beckmann C.F., Auerbach E.J., Douaud G., Sexton C.E., Zsoldos E., Ebmeier K.P., Filippini N., Mackay C.E., Moeller S., Xu J., Yacoub E., Baselli G., Ugurbil K., Miller K.L., Smith S.M. (2014). ICA-based artefact removal and accelerated fMRI acquisition for improved resting state network imaging. NeuroImage.

[bb0175] Huppi P.S., Maier S.E., Peled S., Zientara G.P., Barnes P.D., Jolesz F.A., Volpe J.J. (1998). Microstructural development of human newborn cerebral white matter assessed in vivo by diffusion tensor magnetic resonance imaging. Pediatr. Res..

[bb0180] Inder T.E., Warfield S.K., Wang H., Huppi P.S., Volpe J.J. (2005). Abnormal cerebral structure is present at term in premature infants. Pediatrics.

[bb0185] Kapellou O., Counsell S.J., Kennea N., Dyet L., Saeed N., Stark J., Maalouf E., Duggan P., Ajayi-Obe M., Hajnal J., Allsop J.M., Boardman J., Rutherford M.A., Cowan F., Edwards A.D. (2006). Abnormal cortical development after premature birth shown by altered allometric scaling of brain growth. PLoS Med..

[bb0190] Kiviniemi V., Starck T., Remes J., Long X., Nikkinen J., Haapea M., Veijola J., Moilanen I., Isohanni M., Zang Y.-F., Tervonen O. (2009). Functional segmentation of the brain cortex using high model order group PICA. Hum. Brain Mapp..

[bb0195] Kostovic I., Judas M. (2006). Prolonged coexistence of transient and permanent circuitry elements in the developing cerebral cortex of fetuses and preterm infants. Dev. Med. Child Neurol..

[bb0200] Krzywinski M.I., Schein J.E., Birol I., Connors J., Gascoyne R., Horsman D., Jones S.J., Marra M.A. (2009). Circos: an information aesthetic for comparative genomics. Genome Res..

[bb0205] Kursa M.B., Rudnicki W.R. (2010). Feature selection with the Boruta package. J. Stat. Softw..

[bb0210] Lin W., Zhu Q., Gao W., Chen Y., Toh C.-H., Styner M., Gerig G., Smith J.K., Biswal B., Gilmore J.H. (2008). Functional connectivity MR imaging reveals cortical functional connectivity in the developing brain. AJNR Am. J. Neuroradiol..

[bb0215] Marlow N., Wolke D., Bracewell M.A., Samara M., EPICure Study Group (2005). Neurologic and developmental disability at six years of age after extremely preterm birth. N. Engl. J. Med..

[bb0220] McQuillen P.S., Sheldon R.A., Shatz C.J., Ferriero D.M. (2003). Selective vulnerability of subplate neurons after early neonatal hypoxia-ischemia. J. Neurosci. Off. J. Soc. Neurosci..

[bb0225] Ment L.R., Hirtz D., Huppi P.S. (2009). Imaging biomarkers of outcome in the developing preterm brain. Lancet Neurol..

[bb0230] Moore A.R., Zhou W.-L., Jakovcevski I., Zecevic N., Antic S.D. (2011). Spontaneous electrical activity in the human fetal cortex in vitro. J. Neurosci. Off. J. Soc. Neurosci..

[bb0235] Moore T., Hennessy E.M., Myles J., Johnson S.J., Draper E.S., Costeloe K.L., Marlow N. (2012). Neurological and developmental outcome in extremely preterm children born in England in 1995 and 2006: the EPICure studies. BMJ.

[bb0240] Nichols T. (2013). Notes on creating a standardized version of DVARS.

[bb0245] Padilla N., Alexandrou G., Blennow M., Lagercrantz H., Ådén U. (2014). Brain growth gains and losses in extremely preterm infants at term. Cereb. Cortex.

[bb0250] Pannek K., Hatzigeorgiou X., Colditz P.B., Rose S. (2013). Assessment of structural connectivity in the preterm brain at term equivalent age using diffusion MRI and T2 relaxometry: a network-based analysis. PLoS ONE.

[bb0255] Pedregosa F., Varoquaux G., Gramfort A., Michel V., Thirion B., Grisel O., Blondel M., Prettenhofer P., Weiss R., Dubourg V., Vanderplas J., Passos A., Cournapeau D., Brucher M., Perrot M., Duchesnay É. (2011). Scikit-learn: machine learning in Python. J. Mach. Learn. Res..

[bb0260] Power J.D., Barnes K.A., Snyder A.Z., Schlaggar B.L., Petersen S.E. (2012). Spurious but systematic correlations in functional connectivity MRI networks arise from subject motion. NeuroImage.

[bb0265] Rathbone R., Counsell S.J., Kapellou O., Dyet L., Kennea N., Hajnal J., Allsop J.M., Cowan F., Edwards A.D. (2011). Perinatal cortical growth and childhood neurocognitive abilities. Neurology.

[bb0270] Salimi-Khorshidi G., Douaud G., Beckmann C.F., Glasser M.F., Griffanti L., Smith S.M. (2014). Automatic denoising of functional MRI data: combining independent component analysis and hierarchical fusion of classifiers. NeuroImage.

[bb0275] Satterthwaite T.D., Elliott M.A., Gerraty R.T., Ruparel K., Loughead J., Calkins M.E., Eickhoff S.B., Hakonarson H., Gur R.C., Gur R.E., Wolf D.H. (2013). An improved framework for confound regression and filtering for control of motion artifact in the preprocessing of resting-state functional connectivity data. NeuroImage.

[bb0280] Segovia K.N., McClure M., Moravec M., Luo N.L., Wan Y., Gong X., Riddle A., Craig A., Struve J., Sherman L.S., Back S.A. (2008). Arrested oligodendrocyte lineage maturation in chronic perinatal white matter injury. Ann. Neurol..

[bb0285] Serag A., Aljabar P., Ball G., Counsell S.J., Boardman J.P., Rutherford M.A., Edwards A.D., Hajnal J.V., Rueckert D. (2012). Construction of a consistent high-definition spatio-temporal atlas of the developing brain using adaptive kernel regression. NeuroImage.

[bb0290] Smith S.M., Fox P.T., Miller K.L., Glahn D.C., Fox P.M., Mackay C.E., Filippini N., Watkins K.E., Toro R., Laird A.R., Beckmann C.F. (2009). Correspondence of the brain’s functional architecture during activation and rest. Proc. Natl. Acad. Sci. U. S. A..

[bb0295] Smith S.M., Miller K.L., Salimi-Khorshidi G., Webster M., Beckmann C.F., Nichols T.E., Ramsey J.D., Woolrich M.W. (2011). Network modelling methods for FMRI. NeuroImage.

[bb0300] Smith S.M., Vidaurre D., Beckmann C.F., Glasser M.F., Jenkinson M., Miller K.L., Nichols T.E., Robinson E.C., Salimi-Khorshidi G., Woolrich M.W., Barch D.M., Uğurbil K., Van Essen D.C. (2013). Functional connectomics from resting-state fMRI. Trends Cogn. Sci..

[bb0305] Smyser C.D., Inder T.E., Shimony J.S., Hill J.E., Degnan A.J., Snyder A.Z., Neil J.J. (2010). Longitudinal analysis of neural network development in preterm infants. Cereb. Cortex.

[bb0310] Smyser C.D., Snyder A.Z., Shimony J.S., Mitra A., Inder T.E., Neil J.J. (2014). Resting-state network complexity and magnitude are reduced in prematurely born infants. Cereb. Cortex.

[bb0315] Toulmin H., Beckmann C.F., O’Muircheartaigh J., Ball G., Nongena P., Makropoulos A., Ederies A., Counsell S.J., Kennea N., Arichi T., Tusor N., Rutherford M.A., Azzopardi D., Gonzalez-Cinca N., Hajnal J.V., Edwards A.D. (2015). Specialization and integration of functional thalamocortical connectivity in the human infant. Proc. Natl. Acad. Sci..

[bb0320] Van Dijk K.R.A., Sabuncu M.R., Buckner R.L. (2012). The influence of head motion on intrinsic functional connectivity MRI. NeuroImage.

[bb0325] Vanhatalo S., Kaila K. (2006). Development of neonatal EEG activity: from phenomenology to physiology. Semin. Fetal. Neonatal Med..

[bb0330] Varoquaux G., Craddock R.C. (2013). Learning and comparing functional connectomes across subjects. NeuroImage.

[bb0335] Varoquaux G., Gramfort A., Poline J., Thirion B., Lafferty J.D., Williams C.K.I., Shawe-Taylor J., Zemel R.S., Culotta A. (2010). Brain covariance selection: better individual functional connectivity models using population prior. Advances in Neural Information Processing Systems 23.

[bb0340] Varoquaux, Sadaghiani S., Pinel P., Kleinschmidt A., Poline J.B., Thirion B. (2010). A group model for stable multi-subject ICA on fMRI datasets. NeuroImage.

[bb0345] Vincent J.L., Patel G.H., Fox M.D., Snyder A.Z., Baker J.T., Van Essen D.C., Zempel J.M., Snyder L.H., Corbetta M., Raichle M.E. (2007). Intrinsic functional architecture in the anaesthetized monkey brain. Nature.

[bb0350] Volpe J.J. (2009). Brain injury in premature infants: a complex amalgam of destructive and developmental disturbances. Lancet Neurol..

[bb0355] Wu C.W., Chen C.-L., Liu P.-Y., Chao Y.-P., Biswal B.B., Lin C.-P. (2011). Empirical evaluations of slice-timing, smoothing, and normalization effects in seed-based, resting-state functional magnetic resonance imaging analyses. Brain Connect..

[bb0360] Young J.M., Powell T.L., Morgan B.R., Card D., Lee W., Smith M.L., Sled J.G., Taylor M.J. (2015). Deep grey matter growth predicts neurodevelopmental outcomes in very preterm children. NeuroImage.

[bb0365] Zhang D., Snyder A.Z., Fox M.D., Sansbury M.W., Shimony J.S., Raichle M.E. (2008). Intrinsic functional relations between human cerebral cortex and thalamus. J. Neurophysiol..

