# Supplementary material for: Machine-learning to characterise neonatal functional connectivity in the preterm brain
Source: Neuroimage. 2016 Jan 1;124(Pt A):267–75. doi: 10.1016/j.neuroimage.2015.08.055 (PMC4655920; doi:10.1016/j.neuroimage.2015.08.055)
Supplement: Supplementary file 1 — Supplementary material. [file mmc1.docx]

# Supplemental Material

## Motion assessment

After motion correction via affine registration to a reference volume, motion traces – translation and rotation in 3 planes – were visually analysed. Whole datasets were excluded based on the presence of large reference-to-reference movements (>2mm translation, >2deg rotation in any plane) and/or a mean relative frame-to-frame displacement (MRD) > 0.15mm.

## FIXing neonatal datasets

40 preterm subjects were selected from fMRI datasets acquired on-site for hand-classification and training of the FIX algorithm (Salimi-Khorshidi et al., 2014). Datasets were stratified by age: 20 scanned at or around term-equivalent age (37 to 44 weeks PMA) and 20 scanned during the preterm period between 28 and 36 weeks PMA; within each group 10 were rated as ‘low’ motion and 10 as ‘high’ based on visual assessment of movement traces after motion correction.

Following single-subject ICA, noise-based components were classified by a single rater (G.B.) according to guidelines in (Salimi-Khorshidi et al., 2014) and used to train FIX. Classification accuracy was assessed using leave-one-out bootstrap testing across several classification thresholds. Components were classified using either age-matched or motion-matched (i.e.: all images with high/low motion) training sets. Table S1 shows the classification accuracies of each training set for 10 subjects scanned with relatively low motion at term-equivalent age. As expected, performance was improved when the training dataset comprised subjects of similar age, but accuracy was also improved by including infants with high scanner motion, even when classifying low-motion datasets. The best performance, balancing both true positive and true negative rate was achieved using a full training set of 40 subjects ranging in age and motion. At a classification threshold of 20%, this training set yielded the highest accuracy (average of true positive and true negative rate: 80.1%) across all subjects. The full training dataset (n=40) with a classification threshold of 20% was used to ‘clean-up’ all subjects’ fMRI data in this study.

## Table S1: FIX classification accuracy in ten low-motion datasets acquired at term-equivalent age

|  |  |  |  |  |  |  |  |  |  |  |
| --- | --- | --- | --- | --- | --- | --- | --- | --- | --- | --- |
|  | **Training set** | **FIX classification threshold** | |  |  |  |  |  |  |  |
|  | **True positive rate*** | **1** | **2** | **5** | **10** | **20** | **30** | **40** | **50** |  |
|  | Age- and motion-matched | 98.0 | 96.6 | 95.2 | 92.6 | 86.3 | 79.9 | 71.1 | 69.8 |  |
|  | Age-matched, high motion | 97.4 | 96.8 | 96.2 | 90.9 | 84.3 | 80.8 | 67.5 | 53 |  |
|  | Motion-matched, young age | 94.85 | 93.46 | 92.8 | 87.97 | 83.38 | 83.38 | 77.21 | 69.82 |  |
|  | All | 100 | 98.62 | 97.96 | 96.71 | 94.04 | 90.57 | 86.73 | 84.05 |  |
|  | **True negative rate^#^** |  |  |  |  |  |  |  |  |  |
|  | Age- and motion-matched | 42.4 | 47.8 | 55.1 | 62.9 | 73.3 | 82.4 | 86.7 | 90.8 |  |
|  | Age-matched, high motion | 30.5 | 35.67 | 48.01 | 62.31 | 72.6 | 79.46 | 87.1 | 91.57 |  |
|  | Motion-matched, young age | 50.39 | 54.43 | 58.95 | 64.28 | 74.06 | 81.17 | 86.75 | 90.04 |  |
|  | All | 35.88 | 42.48 | 49.2 | 56.51 | 66.19 | 73.44 | 77.94 | 82 |  |
|  | **Accuracy^+^** |  |  |  |  |  |  |  |  |  |
|  | Age- and motion-matched | 70.2 | 72.2 | 75.1 | 77.7 | 79.8 | 81.1 | 78.9 | 80.3 |  |
|  | Age-matched, high motion | 64.0 | 66.2 | 72.1 | 76.6 | 78.5 | 80.1 | 77.3 | 72.3 |  |
|  | Motion-matched, young age | 72.6 | 73.9 | 75.9 | 76.1 | 78.7 | 82.3 | 82.0 | 79.9 |  |
|  | All | 67.9 | 70.6 | 73.6 | 76.6 | 80.1 | 82.0 | 82.3 | 83.0 |  |
|  | * % components rated as signal correctly identified by FIX | | | |  |  |  |  |  |  |
|  | ^#^ % components rated as noise correctly identified by FIX | | |  |  |  |  |  |  |  |
|  | ^+^ Average of true positive and true negative rates | | |  |  |  |  |  |  |  |
